# An explorative, cross-sectional study into abnormal muscular coupling during reach in chronic stroke patients

**DOI:** 10.1186/1743-0003-7-14

**Published:** 2010-03-16

**Authors:** Gerdienke B Prange, Michiel JA Jannink, Arno HA Stienen, Herman van der Kooij, Maarten J IJzerman, Hermie J Hermens

**Affiliations:** 1 Roessingh Research & Development, Roessinghsbleekweg 33b, Enschede, the Netherlands; 2 University of Twente, Department of Biomechanical Engineering, Drienerlolaan 5, Enschede, the Netherlands; 3 Northwestern University, Department of Physical Therapy & Human Movement Science, 645 North Michigan Avenue, Chicago (IL), USA; 4 Delft University of Technology, Department of Biomechanical Engineering, Stevinweg 1, Delft, the Netherlands; 5 University of Twente, Department of Health Technology & Services Research, Drienerlolaan 5, Enschede, the Netherlands; 6 University of Twente, Department of Electrical Engineering, Mathematics and Computer Science, Drienerlolaan 5, Enschede, the Netherlands

## Abstract

**Background:**

In many stroke patients arm function is limited, which can be related to an abnormal coupling between shoulder and elbow joints. The extent to which this can be translated to activities of daily life (ADL), in terms of muscle activation during ADL-like movements, is rather unknown. Therefore, the present study examined the occurrence of abnormal coupling on functional, ADL-like reaching movements of chronic stroke patients by comparison with healthy persons.

**Methods:**

Upward multi-joint reaching movements (20 repetitions at a self-selected speed to resemble ADL) were compared in two conditions: once facilitated by arm weight compensation and once resisted to provoke a potential abnormal coupling. Changes in movement performance (joint angles) and muscle activation (amplitude of activity and co-activation) between conditions were compared between healthy persons and stroke patients using a repeated measures ANOVA.

**Results:**

The present study showed slight changes in joint excursion and muscle activation of stroke patients due to shoulder elevation resistance during functional reach. Remarkably, in healthy persons similar changes were observed. Even the results of a sub-group of the more impaired stroke patients did not point to an abnormal coupling between shoulder elevation and elbow flexion during functional reach.

**Conclusions:**

The present findings suggest that in mildly and moderately affected chronic stroke patients ADL-like arm movements are not substantially affected by abnormal synergistic coupling. In this case, it is implied that other major contributors to limitations in functional use of the arm should be identified and targeted individually in rehabilitation, to improve use of the arm in activities of daily living.

## Background

After stroke, limitations in arm function are common [1], with varying sensory and motor symptoms, all contributing to a reduced ability to coordinate movements [2]. This can, amongst others, be expressed as an involuntary coupling of movements, as was already recognized by Brunnstrom several decades ago [3]. She distinguished two patterns of coupling to describe the motor behavior of stroke patients: a flexion pattern and an extension pattern. For the upper extremity, the flexion pattern includes shoulder abduction, shoulder external rotation, elbow flexion and forearm supination, while the extension pattern comprises shoulder adduction, shoulder internal rotation, elbow extension and forearm pronation.

Beer, Dewald and colleagues showed that in isometric contractions of chronic stroke patients the generation of shoulder abduction torques is coupled to simultaneous generation of elbow flexion torques: the higher the shoulder abduction torque, the more elbow flexion is generated [4,5]. When extending this research to dynamic situations, they found limitations in elbow extension during reaching without arm support when the arm has to be lifted actively at shoulder height, since active shoulder abduction provoked simultaneous elbow flexion torques [6].

Besides this insight into kine(ma)tics of abnormal coupling in stroke, only some information is available about the muscle activation patterns during this abnormal coupling. Dewald et al. indicated that activity of the shoulder abducting muscles, deltoid and upper trapezius, is correlated to elbow flexor muscles and that the shoulder adducting pectoral muscle is activated concurrently with elbow extensor muscles during isometric torque generation, while the affected arm of chronic stroke patients is held at shoulder height [4]. These findings indicate that the flexion and extension patterns are also expressed in muscle activity during simultaneous isometric contractions of shoulder and elbow muscles after stroke.

In the abovementioned studies, abnormal coupling between shoulder abduction and elbow flexion was identified during reaching movements with the arm in a position not frequently encountered during activities of daily living, i.e., with the upper arm held at or near shoulder height throughout the reaching movement. However, it is not known whether these findings can be extended to actual reaching movements corresponding with functional movements as applied in everyday life. Such movements, often starting at table height, require less shoulder abduction. Research has shown that the impact of abnormal coupling reduces with decreasing shoulder abduction torque [5,7]. It is unclear how these characteristics affect activities of daily living (ADL) and what potential consequences are for clinical practice.

This study was designed to examine the occurrence of abnormal muscular coupling during functional, ADL-like reaching movements of chronic stroke patients at the level of muscle activation and movement kinematics. For this purpose, upward multi-joint reaching movements starting at table height were compared between two conditions: once reaching was facilitated by gravity compensation and once reaching was resisted to provoke a potential involuntary coupling. To identify abnormal patterns of coupling, changes in muscle activation and kinematics between both conditions were assessed. These differences were then compared between chronic stroke patients and healthy persons. It was expected that in chronic stroke patients an increased generation of shoulder elevation torques during resisted reach is accompanied by a more pronounced reduction in elbow extension and an increased coupling of shoulder abductor and elbow flexor muscles, compared with healthy persons.

## Methods

### Subjects

A random sample of 15 chronic stroke patients, receiving or having received care from a local rehabilitation centre, was selected. Participants had to meet the following inclusion criteria: 1) age between 25 and 75 years; 2) at least 6 months post-stroke; 3) ability to lift the arm (at least partly) against gravity, without full recovery of selective shoulder and elbow movements; 4) no pain or other condition interfering with the mobility and/or strength of the arm; 5) ability to understand and follow instructions; 6) provide written informed consent.

Five healthy persons with no history of arm function impairments were included to compare findings in chronic stroke patients with unimpaired movement control and performance. The study was approved by the local medical ethics committee (METC of Rehabilitation Center 'Het Roessingh', Enschede, the Netherlands).

### Procedure

Movement ability and reach performance (with and without resistance) of subjects was assessed on 1 occasion. The upper extremity portion of the Fugl-Meyer assessment (FM) was performed by the stroke patients to document the status of motor recovery and arm function of the hemiparetic arm [8]. This measure was used as a description of the motor status of the included stroke patients at the time of the study.

**Figure 1:**
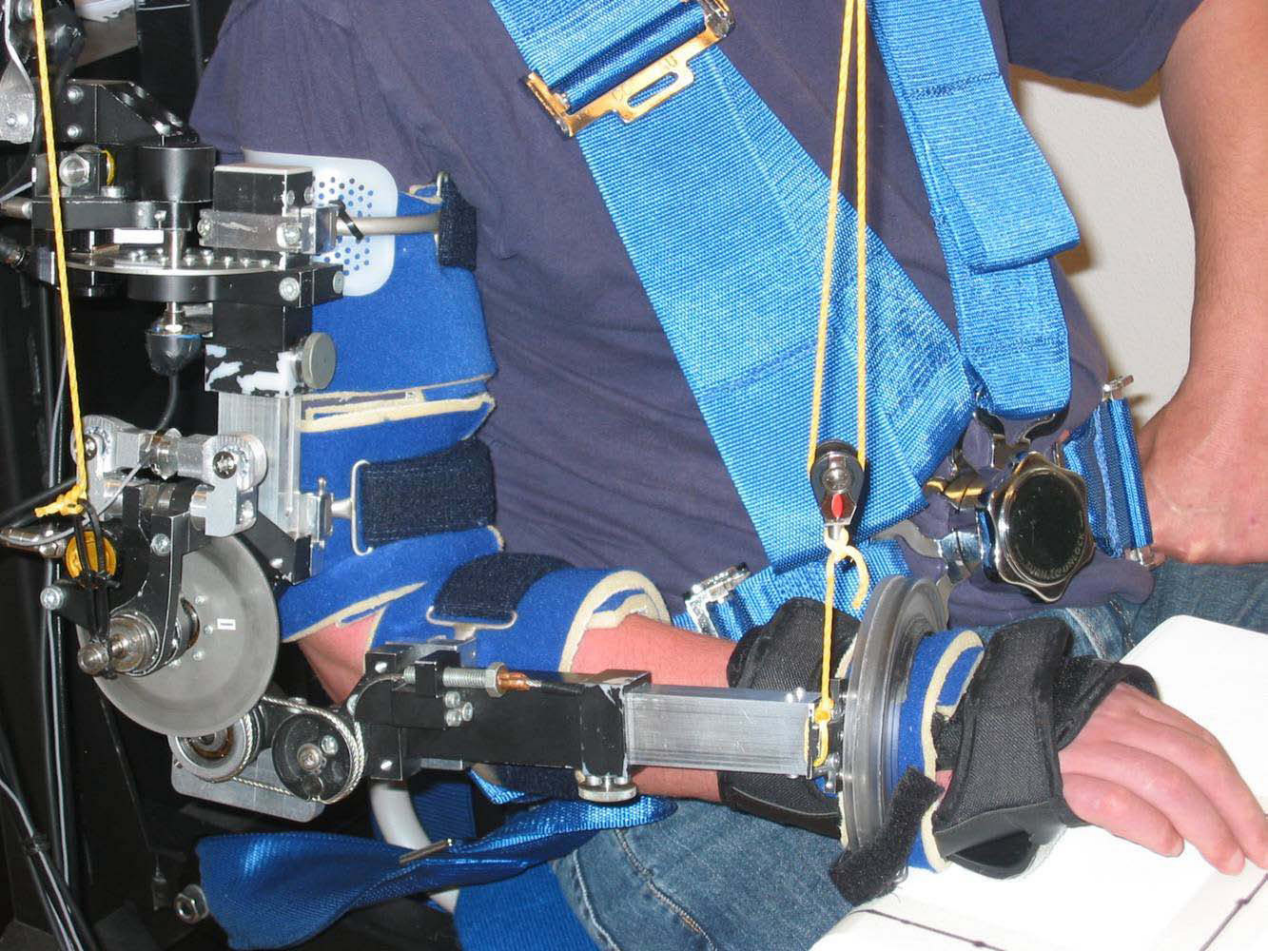
**Dampace exoskeleton for joint-specific resistance**.

During the reaching task, subjects were seated with straps over the trunk to limit compensational trunk movements, with the upper arm aligned with the trunk (shoulder in 0° anteflexion and 0° abduction) and the elbow flexed 90° (figure 1). The wrist was placed in a position as neutral as possible by fixation to a splint (midway between flexion/extension and radial/ulnar abduction) and the hand was balled to a fist as much as possible. A starting square of 10 × 10 cm was placed under the subject's hand and the target square of 10 × 10 cm was placed just below shoulder height at 90% of the subject's active range of motion, at an angle of approximately 30° lateral from the sagittal plane at the shoulder. This resulted in upward and outward reaching movements using both shoulder and elbow rotations, resembling for instance reaching for a cup in a drawer.

Two sets of multi-joint reaching movements (20 repetitions in each set) were performed. Once arm elevation was facilitated by compensating the weight of the arm, once arm elevation was resisted at the shoulder. The reaching movements were performed at a self-selected speed, to match functional use of the arm as much as possible. The order of the conditions (with and without resistance) was randomized across participants (using a table of random numbers), to limit any potential influence of fatigue or adaptation. Besides this, subjects got accustomed to the experimental set-up by repeating the required movement several times prior to data recording.

### Application of resistance

Arm weight compensation and joint-specific resistance was applied to alter the shoulder elevation torque during reach, with the use of an exoskeleton (Dampace, see figure 1) [9]. Three degrees of freedom at the shoulder enable transversal rotation (corresponding with *horizontal abduction*), elevation (which corresponds with shoulder *abduction and/or anteflexion *expressed within the clinical framework), and axial rotation (corresponding with *endo-/exorotation*) of the upper arm. One degree of freedom at the elbow enables flexion/extension and a flexible wrist attachment allows pro-/supination of the forearm. The exoskeleton is attached to a rigid frame, situated behind the subject, in such a way that the shoulder and scapula can move freely. More details of the Dampace can be found in Stienen et al. 2007 [9] and Stienen et al. 2009 [10].

The gravitational pull on the exoskeleton was compensated by a system of ideal springs, attached to the exoskeleton by wires via several pulleys overhead. Although this does not eliminate inertial effects of the exoskeleton, application of low movement speeds, as in the current experiment, render the inertial forces negligible. To facilitate reaching movements in one condition, this system was set to provide compensation of 100% of a person's arm weight. In the other condition, specific resistance torques were applied to the shoulder elevation axis by a hydraulic disc brake. The braking force was controlled by a computer, based on measurements of integrated position and torque sensors. The amount of resistance was set to 80% of the shoulder elevation torque needed to lift the arm. In healthy persons, this level of resistance corresponded with 11 to 19% of their maximal voluntary shoulder elevation torque across subjects, in stroke patients this was 23 to 65%.

### Measurements

During reach, muscle activity and kinematics were recorded.

#### Muscle activity

Bi-polar surface electromyography (EMG) was recorded from 8 upper extremity muscles using Ag/AgCl-electrodes (Neuroline, type 720 00-S; Medicotest A/S, Ølstykke, Denmark), according to the guidelines of the SENIAM project [11]. The EMG signals of biceps (BIC), brachioradialis (BRA), long and lateral head of triceps (TILO and TILA), anterior deltoid (AD), posterior deltoid (PD), lattissimus dorsi (LD) and upper trapezius (TRA) were measured and amplified using a 16-channel Porti system (Twente Medical Systems International, Oldenzaal, the Netherlands) and digitized by a 22-bit analog-to-digital converter with a sample rate of 1024 samples per second and stored on a computer. For real-time display, the EMG signals were high-pass filtered (3^rd ^order Butterworth filter, cut-off frequency 5 Hz) during the measurements. The recorded EMG signals were, off-line, band-pass filtered (2^nd ^order zero phase shift Butterworth, cut-off frequencies 10-400 Hz) and converted to smooth rectified EMG (SRE) signals (using a low-pass 2^nd ^order zero phase shift Butterworth filter at 25 Hz for smoothing).

#### Kinematics

Kinematic data of arm segments were recorded using integrated position sensors in the Dampace at each movement axis of the shoulder and elbow. The voltages over the potentiometers at the shoulder axes were converted from analog to digital values by a DAQ card (National Instruments, Austin, Texas) with a sample rate of 1000 Hz. The elbow angle is measured by an integrated two-channel rotational optical encoder (US Digital, Vancouver, Washington). The elbow joint angle was specified as the angle between humerus and forearm (maximal elbow extension is 180°). The shoulder joint orientation was described using two angles according to recommendations of the International Society of Biomechanics [12]. The plane of elevation (*transversal rotation *or *horizontal abduction*) was defined as the angle of the humerus with a virtual line through both shoulders, viewed in the transversal plane (outward/lateral is 0°; arm extended forward is 90°). The angle of elevation (shoulder *abduction and/or anteflexion*) was the angle between humerus and trunk in the plane of elevation (consisting of the vertical plane through the upper arm), irrespective of the orientation of the humerus in the transversal plane (humerus parallel with trunk is 0°, humerus horizontal is 90°). These data were real-time filtered with a first order Butterworth low-pass filter with a cut-off frequency of 40 Hz. Filtering was performed in a Simulink model (The Mathworks Inc, Natick, Massachusetts) which was compiled into an executable using the RealTime Application Interface for Linux http://www.rtai.org. Measured signals were stored on a computer with a sample frequency of 50 Hz. Off-line, the kinematic data were linearly interpolated from 50 to 1024 Hz to match the sample rate of the EMG recordings.

### Data analysis

The SRE signals and joint angles were synchronized and averaged over all repeated reaching movements within both sets of 20 repetitions (with and without resistance). Start and end of reaching movements were defined by the elbow joint angle, with the minimum angle representing the start of reach and the maximum angle representing the end of reach. The duration of the reaching movement was expressed as 100%, to account for intra- and inter-subject variation.

Analysis comprised initial qualitative inspection of muscle activation patterns and subsequent calculation of quantitative measures. The level of muscle activity was represented by the mean SRE-value during the averaged reaching movement. To evaluate relative changes in the contribution of each muscle to reach within each subject, the SRE-value of each muscle was related to the sum of SRE-values recorded from the 8 muscles (input%; percentage of mean SRE-value of each muscle with respect to the cumulative SRE-value of all 8 muscles per subject). Additional information about inter-muscle coupling in each subject was provided by individually calculating the ratio between the average SRE-values of elbow flexors (BIC and BRA) and the shoulder elevator (AD) so that co-contraction ratios (CCratios) of BIC and AD and of BRA and AD were obtained. Additionally, ratios between BIC and TRA, and TILA/TILO and AD were calculated.

To quantify movement performance, movement time (in ms), minimal (i.e., at the start of reach), maximal (i.e., at the end of reach) angles (in °) of shoulder and elbow joints and the difference between minimal and maximal joint angles (i.e., joint excursion or range of motion) were calculated for each averaged reaching movement. The changes in outcome measures between reaching movements with and without shoulder elevation resistance (SE-resistance) were compared between healthy subjects and chronic stroke patients.

### Statistical analysis

All outcome measures were inspected for normal distribution of data using histogram plots including normal curves and normal probability plots prior to selection of proper statistical tests. Differences in movement time between movements with and without SE-resistance in both healthy persons and chronic stroke patients were tested using a paired samples t-test, or its non-parametric equivalent (Wilcoxon signed ranks test). Minimal (min), maximal (max) and range of motion (ROM) values of all joint angles were compared between movements with and without SE-resistance (within-subjects factors of 'resistance', 'joint' and 'gonio') and between healthy persons and chronic stroke patients (between-subjects factor of 'status') using analysis of variance (ANOVA) for repeated measures.

SRE-values and CCratios were log-transformed prior to statistical analysis to ensure normal distribution of the data. For each muscle, mean SRE-values and input% were compared between movements with and without SE-resistance and between healthy persons and chronic stroke patients using an ANOVA for repeated measures, using a within-subjects factor for 'resistance' (with or without SE-resistance) and a between-subjects factor for 'status' (healthy or stroke). The same procedure was repeated for the CCratios.

To assess potential differences in the occurrence of abnormal coupling during functional reach between stroke patients with varying stroke severity, additional analyses have been performed using a similar repeated measures ANOVA as mentioned above. In this case, the between-subjects factor was not 'status' with 2 levels (healthy and stroke), but 'stroke severity' with 3 levels (unimpaired, mild and moderate hemiparesis). The division between mild and moderate stroke patients was based on the Fugl-Meyer scale: a score above 45 points was regarded as mild hemiparesis; a score between 20 and 45 points was regarded as moderate hemiparesis. Below 20 points should be considered severely affected, but these subjects were not included in this study. For all tests, the significance level was defined as 0.05.

## Results

### Subjects

One of the 15 included stroke patients was not able to complete the tasks due to severe fatigue. The data of a second stroke patient was not complete due to technical problems during the measurements. The data of these 2 subjects were excluded from data analysis. Data of 5 healthy persons (4 male) and 13 stroke patients (9 male) was available for analysis (see table 1 for details). All stroke patients were in the chronic phase, with the time post-stroke varying from 7 to 126 months. The level of arm function, as measured by the Fugl Meyer assessment (FM), ranged from 22 to 65, with an average score of 51 points. Of the 13 stroke patients, 9 had FM scores larger than 45 points (regarded as mild hemiparesis), whereas 4 patients had FM scores between 20 and 45 points (regarded as moderate hemiparesis).

**Table 1:** Descriptive (mean ± SD) subject characteristics

	*Healthy subjects (n = 5)*	*Stroke patients (n = 13)*
*Age (years)*	54.4 (± 19.0)	65.9 (± 6.9)
*Body mass index (kg/m*^2^)	22.5 (± 0.99)	25.5 (± 3.6)
*Time post-stroke (months)*	not applicable	26 (± 31)
*FM score (points of max 66)*	not applicable	51 (± 13)

### Movement performance

Mean movement time did not differ significantly between movements with and without SE-resistance in healthy persons and chronic stroke patients (p ≥ 0.510). Since the movement amplitude was fixed, this indicates no difference in movement speed. When comparing both groups, chronic stroke patients showed somewhat larger movement times than healthy persons (respectively 1.3 s and 0.9 s, p ≤ 0.034).

**Figure 2:**
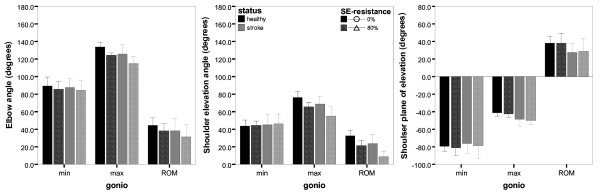
**Mean (± SD) of extremes and ranges of joint angles with and without SE-resistance**. Different panels display elbow flexion/extension (left), shoulder angle of elevation (middle) and shoulder plane of elevation (right) in healthy persons (black bars) and chronic stroke patients (grey bars) per resistance condition (0% and 80% in solid and striped bars, resp.)

Mean joint angle extremes and ranges of healthy persons and stroke patients are displayed in figure 2 per condition. Inspection of these data showed that in both groups angles and excursions of several joints decreased with resistance. Maximal elbow (E) and shoulder elevation (SE) angles (at the end of reach) and their excursions (ROM) were 7° to 14° smaller with resistance ('resistance' p ≤ 0.004). Although this led to SE angles smaller than 5° in 5 of the 13 stroke patients, all subjects (both healthy persons and stroke patients) could still reach the target at shoulder height with resistance. Minimal angles (at the start of reach) were similar in both conditions, except for the minimal E-angle, which was slightly smaller (i.e., the elbow was more flexed) with an average of 3° at the start of resisted reach (p = 0.015). The shoulder plane of elevation (SP) remained largely unchanged.

Despite these changes within subjects, no significant differences were found between healthy persons and chronic stroke patients. Concerning sub-group analysis, a small trend towards larger limitations in maximal E-angles with SE-resistance in moderately affected stroke patients was observed compared with unimpaired persons. Nevertheless, these changes with resistance were not significantly different between sub-groups of stroke severity (mild stroke vs. moderate stroke vs. unimpaired groups), as found in additional analyses (p ≥ 0.541).

### Muscle activation

To examine the expression of any abnormal coupling between muscles in chronic stroke patients, we compared changes in muscle activity due to the application of SE-resistance between healthy persons and stroke patients.

#### Muscle activity levels

**Figure 3:**
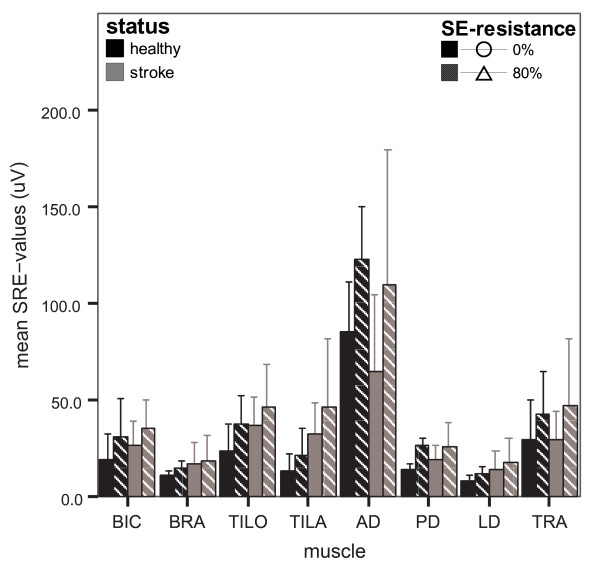
**Mean (± SD) SRE-values per muscle with and without SE-resistance**. Data of healthy persons are displayed in black bars and of chronic stroke patients in grey bars per resistance condition (0% and 80% in solid and striped bars, resp.)

With respect to movements without SE-resistance, the activity of all muscles increased during movements with SE-resistance, in both healthy persons and chronic stroke patients (figure 3). The increases of AD, TRA and, to a smaller extent, BIC reflect the enhanced SE-torque to be generated with resistance. This slightly increased BIC activity requires some increase in activity of the elbow extensor muscles (TILO and TILA) to achieve the reaching task. In addition, it is likely that with SE-resistance more stabilization of the shoulder joint is needed to control the larger shoulder elevation forces, resulting in slightly increased activity levels of PD and LD. These increases were significant in all muscles ('resistance' p ≤ 0.007).

When comparing healthy persons and chronic stroke patients, few differences in SRE-values were found between both groups ('status' p ≥ 0.176). One of the differences concerned TILA, in which overall SRE-values were higher in chronic stroke patients compared to healthy persons (p = 0.019). The increases in SRE-values with SE-resistance did not differ significantly between healthy persons and chronic stroke patients in most muscles ('status × resistance' p ≥ 0.082), except for TILO (p = 0.019) and PD (p = 0.009). In those two muscles, the increases in SRE-values with resistance were somewhat smaller in chronic stroke patients than in healthy persons.

An enhanced expression of abnormal flexion coupling should result in more pronounced increases in SRE-values with resistance in chronic stroke patients than in healthy persons, especially regarding shoulder elevators and elbow flexors. However, the findings do not support this expectation. As with joint angle data, this was not different when examining sub-groups of stroke severity (mild stroke vs. moderate stroke vs. unimpaired groups) in additional analyses. However, a small trend was observed on visual inspection of data towards a higher activity level of BIC and a more pronounced decrease in AD activity with resistance in moderately affected stroke patients compared with unimpaired persons. Also, PD activity was more pronounced and LD activity was less pronounced in moderately affected stroke patients compared with unimpaired persons.

#### Contribution of individual muscles to reach

**Figure 4:**
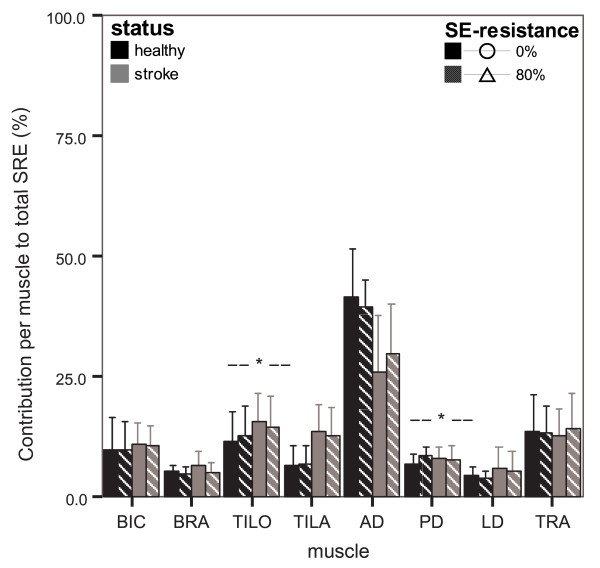
**Mean (± SD) of relative muscle contributions (input%) with and without SE-resistance**. Data of healthy persons are displayed in black bars and of chronic stroke patients in grey bars per resistance condition (0% and 80% in solid and striped bars, resp.); asterisks indicate significant differences in changes due to SE-resistance between healthy persons and chronic stroke patients.

When looking at the contribution of each muscle to reach within each subject (input%), it is observed that the application of SE-resistance hardly changed the distribution of input% between muscles (figure 4). Only input% of BRA decreased somewhat when SE-resistance was applied ('resistance' p = 0.014), over all subjects.

Few differences were found between healthy persons and chronic stroke patients. The input% of AD was smaller and the input% of TILA was larger in chronic stroke patients than in healthy persons ('status' p ≤ 0.034). Changes in input% with SE-resistance only differed slightly between healthy persons and chronic stroke patients in TILO and PD. With SE-resistance, input% of TILO decreased in chronic stroke patients, whereas it did not change in healthy persons ('status × resistance' p = 0.032). In PD, input% increased with SE-resistance in healthy persons, whereas no significant change was detected in chronic stroke patients ('status × resistance' p = 0.011).

Although the changes in muscle contributions with resistance were slightly different for two muscles between healthy persons and chronic stroke patients, these differences were not consistent with an increased coupling between S-elevators and E-flexors after stroke. Again, this observation did not alter when regarding sub-groups of stroke severity.

#### Co-contraction of shoulder and elbow muscles

Additional information about specific inter-muscle coupling within each subject was obtained by relating the individual average SRE-values of elbow flexors (BIC and BRA) to the prime mover for the shoulder during the reaching task (AD).

**Figure 5:**
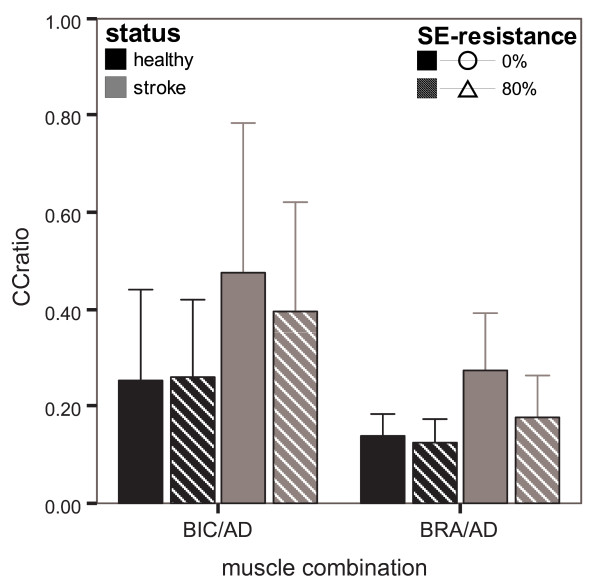
**Mean (± SD) co-contraction ratios of shoulder elevators and elbow flexors**. Data of ratios of BIC to AD and BRA to AD are displayed for healthy persons (black bars) and chronic stroke patients (grey bars) per resistance condition (0% and 80% in solid and striped bars, resp.)

When comparing the values of the mean CCratio of BIC and AD between healthy persons and chronic stroke patients (figure 5), we found that the CCratio remained largely unchanged with resistance ('resistance' p = 0.557) in both healthy persons and chronic stroke patients ('status × resistance' p = 0.379). Overall, the CCratio did not differ significantly between healthy persons and chronic stroke patients ('status' p = 0.091).

The differences in co-contraction of BRA and AD (figure 5) with SE-resistance and between groups are comparable to those of BIC and AD. Statistically, differences were slightly more pronounced, due to a smaller variation in CCratio for BRA and AD across subjects. With SE-resistance, the CCratio decreased significantly when looking overall over both groups ('resistance' p = 0.011). When comparing the CCratios of BRA and AD for healthy persons and chronic stroke patients, no significant differences were observed ('status' p = 0.114). Also, the decreases with resistance were not significantly different between healthy persons and chronic stroke patients ('status × resistance' p = 0.094).

Again, these results do not point to an abnormal coupling between AD and elbow flexors, since an increase in AD activity was accompanied by a less than proportional increase in BIC and BRA activity in both healthy persons and chronic stroke patients. When dividing the stroke patients in sub-groups displaying mild and moderate hemiparesis, the change in CCratio of BIC and AD with resistance was slightly more pronounced ('resistance' p = 0.028), specifically in the moderate group, then when regarding all stroke patients. Nevertheless, the CCratio decreased with resistance, which does not correspond with an increased abnormal coupling between shoulder elevation and elbow flexion, leading to similar conclusions. Also, when regarding additional combinations of other shoulder and elbow muscles, TRA with BIC, and AD with both heads of triceps, this observation did not change.

## Discussion

To examine the occurrence of abnormal, involuntary muscular coupling during functional reaching movements of chronic stroke patients, the present study compared changes in movement execution and muscle activation between ADL-like, multi-joint reaches with and without shoulder elevation resistance at a comfortable speed between healthy persons and chronic stroke patients. The term shoulder elevation as defined in the present study corresponds with shoulder *abduction and/or anteflexion *as commonly used in clinical practice.

The present study showed slight changes in joint excursion and muscle activity of stroke patients due to shoulder elevation resistance during functional reach. Remarkably, similar changes were observed in healthy persons. All subjects were able to reach the target in both conditions. However, in 5 out of 13 stroke patients shoulder elevation excursion was reduced to less than 5° with resistance, making it more difficult to detect a potential abnormal flexion pattern if it were present. Nonetheless, the chronic stroke patients in the present study managed the added resistance in a similar way as healthy persons.

In addition, the increases in muscle activation level with shoulder elevation resistance, observed in all muscles, were comparable between healthy persons and chronic stroke patients. No indications were found that an increase in AD activity was accompanied by a larger increase in elbow flexor activity in chronic stroke patients compared to healthy persons.

Remarkably, even the results of a sub-group of the more impaired stroke patients included in this study, who all displayed abnormal coupling on corresponding items of the FM assessment, did not point to an abnormal coupling between shoulder elevation and elbow flexion during functional reach. The ability to reach was not substantially limited or prevented due to abnormal coupling between shoulder elevation and elbow flexion after stroke. Moreover, the response to movements with resistance in stroke patients was remarkably similar to healthy persons.

In both static and dynamic situations, Beer et al. did identify an involuntary coupling of shoulder elevation torques to simultaneous generation of elbow flexion torques in chronic stroke patients, resulting in reduced elbow extension ability [5,6]. Dewald et al. showed that activity of shoulder abducting muscles is correlated with activity of elbow flexor muscles during isometric torque generation by chronic stroke patients [4]. It is possible that part of the discrepancy between these studies and our study is related to differences in stroke severity of the participants in both studies. The chronic stroke patients included in the present study varied in severity of hemiparesis from patients who could just lift their own arm (FM score of 22) to patients who experienced very few limitations in arm function (FM score of 65), although the majority of stroke patients (9 out of 13 patients) displayed mild hemiparesis. The research by Beer, Dewald and colleagues involved chronic stroke patients with a more severe arm paresis; FM scores ranged from 15 to 60 points in initial research [4], and were even lower in later work with FM scores ranging from 15 to 40 points [13].

Differences concerning the arm position during dynamic evaluations of abnormal coupling are also a plausible cause for the discrepancy. In the research of Beer, Dewald and colleagues, dynamic tasks required an arm position of 75° up to 90° of shoulder abduction during the entire movement task [6,14-16]. The occurrence of the coupling between shoulder abduction and elbow flexion was found to be dependent on the magnitude of generated torques in both static [5] and dynamic situations [7]. This indicates that in the present study, applying smaller shoulder abduction angles during (initiation of) functional reach, a potential coupling would be less prominent, which partly supports our findings.

On the other hand, another study observed that in an isometric situation an abnormal coupling between shoulder abduction and elbow flexion was present with the upper arm positioned in either 70° or 20° of shoulder abduction [17]. McCrea et al. investigated reaching strategies of chronic stroke patients applying a more functional movement of sagittal forward and upward reach, which is comparable to the reaching movement in the present study in terms of required (initial) shoulder elevation [18]. They also did observe an abnormal coupling between shoulder and elbow movements: limitations in shoulder flexion were accompanied by increased shoulder abduction and increased elbow flexion [18].

Besides stroke severity and arm position, differences in movement speed may also play a role in the occurrence of abnormal coupling between shoulder elevation and elbow flexion. In the reaching tasks used to study abnormal coupling in dynamic situations in before-mentioned studies of Beer, Dewald and colleagues, subjects were instructed to move as rapidly as possible [6,14-16]. Also the study by McCrea et al. applied maximal movement speed [18]. In the present study, movement speed was lower by asking subjects to move at a comfortable, self-selected speed, to resemble most movements in daily life. Besides a potential influence of hyper reflexivity and spasticity during very fast movements, a high movement speed poses a larger strain on the neuromuscular system than the movement task in the present study, which may elicit a more pronounced abnormal synergistic coupling.

Remarkable in this context is that reductions in elbow extension with increasing shoulder abduction torques have been observed even during slow arm movements [7]. Then again, this study involved an arm position of 90° shoulder abduction, requiring larger shoulder abduction torques throughout the movement task than the present study involving a more functional arm movement.

Considering the findings of the present study in the context of above-mentioned research, it is plausible that abnormal coupling between shoulder and elbow movements in chronic stroke patients only limits movement performance substantially when a strenuous task has to be performed, either with near-maximal force or with near-maximal speed, or both. The present findings suggest that during sub-maximal, functional movements at lower velocities as encountered in daily life, abnormal coupling between shoulder and elbow movements is not predominant in either movement execution or muscle activation in mildly and moderately affected chronic stroke patients. This is in line with findings that only 13% of the stroke population display abnormal limb synergies at 3 months post-stroke [19].

The present study suggests that in mildly and moderately affected chronic stroke patients, an involuntary coupling, especially between shoulder elevation and elbow flexion, is not a major factor in limitations of functional reach. These findings have to be interpreted with care. This explorative study is based on a limited number of participants, with a relatively high residual arm function. Also, even though visual inspection of SRE traces did not reveal any substantial changes in temporal aspects of muscle activation with resistance, more subtle changes in the temporal aspects of muscle activation may not have been detected. During a tracking task where the arm was fully supported in a 2D plane, differences in timing of peak muscle activation of predominantly triceps, anterior deltoid and upper trapezius between chronic stroke patients and healthy persons have been observed, in addition to a higher amplitude of biceps [20,21]. This indicates that temporal differences and an increased elbow flexor activity may be involved in altered motor control after stroke, depending on movement task (as put forward above) and the applied muscle activation analyses. Furthermore, the stroke patients were older than the healthy persons in the present study. Since control of multi-joint arm movements changes with age, such as a reduction in modulation of amplitude of muscle activation [22], differences in age may have influenced the ability to detect differences in muscle activation between stroke patients and healthy persons in the present study. Nevertheless, in the context of discussed literature that partly supports our findings, more detailed research into the extent to which abnormal coupling between the shoulder and elbow influences functional use of the arm is justified.

For the group of stroke patients whose ability to perform functional arm movements is not restricted by abnormal coupling, interventions aimed at reducing such abnormal movement patterns may not be the most suitable method to improve arm function. In those cases, it would be valuable to asses which impairments do contribute to limitations in arm function. For instance, several studies have identified muscle weakness as a more important factor in limitations in reach performance [23,24] or general arm function [25,26], than a loss of movement selectivity. Identification of such major contributors to impaired arm function may then serve as starting point to choose the optimal rehabilitation strategy.

## Conclusions

The present findings suggest that in mildly and moderately affected chronic stroke patients functional, ADL-like arm movements at comfortable movement speed are not affected by abnormal coupling between shoulder and elbow movements. Even though interpreted carefully, the present study, in the context of previous research, indicates that involuntary, abnormal coupling of shoulder and elbow movements is not predominant in chronic stroke patients with mild to moderate hemiparesis. It is plausible that such abnormal coupling is only evident in a relatively small group of stroke patients with severe hemiparesis, where task demands of ADL-like movements are high enough to reach a certain threshold of physical effort. In stroke patients whose arm function is not substantially limited by abnormal coupling, interventions aimed at reducing such abnormal movement patterns may not be the most suitable method to improve arm function. This implies that the major contributors to limitations in functional use of the arm should be identified and targeted individually in rehabilitation, to improve use of the arm in activities of daily living.

## Competing interests

The authors declare that they have no competing interests.

## Authors' contributions

GP performed the design of the study, acquisition and analysis of data and drafting of the manuscript. MJ made substantial contributions to the design, interpretation of the data and drafting of the manuscript. AS made substantial contributions to acquisition and analysis of the data and to revision of the manuscript. HK, MY and HH were involved in conception and design of the study, interpretation of the data and critical revision of the manuscript for important intellectual content. All authors have read and approved the final manuscript.
